# A Focused Insight into Thyme: Biological, Chemical, and Therapeutic Properties of an Indigenous Mediterranean Herb

**DOI:** 10.3390/nu14102104

**Published:** 2022-05-18

**Authors:** Dalal Hammoudi Halat, Maha Krayem, Sanaa Khaled, Samar Younes

**Affiliations:** 1 Department of Pharmaceutical Sciences, School of Pharmacy, Lebanese International University, Bekaa Campus, Bekaa P.O. Box 146404, Lebanon; 2 Department of Biological and Chemical Sciences, School of Arts and Sciences, Lebanese International University, Bekaa Campus, Bekaa P.O. Box 146404, Lebanon; maha.krayem@liu.edu.lb (M.K.); sanaa.khaled@liu.edu.lb (S.K.); 3 Department of Biomedical Sciences, School of Pharmacy, Lebanese International University, Bekaa Campus, Bekaa P.O. Box 146404, Lebanon; samar.younes@liu.edu.lb

**Keywords:** *Thymus vulgaris*, thyme essential oil, thymol, carvacrol, antioxidant, anti-inflammatory, anticancer, antimicrobial

## Abstract

A perennial wild shrub from the *Lamiaceae* family and native to the Mediterranean region, thyme is considered an important wild edible plant studied for centuries for its unique importance in the food, pharmaceutical, and cosmetic industry. Thyme is loaded with phytonutrients, minerals and vitamins. It is pungent in taste, yet rich in moisture, proteins, crude fiber, minerals and vitamins. Its chemical composition may vary with geographical location but is mainly composed of flavonoids and antioxidants. Previous studies have illustrated the therapeutic effects of thyme and its essential oils, especially thymol and carvacrol, against various diseases. This is attributed to its multi-pharmacological properties that include, but are not limited to, antioxidant, anti-inflammatory, and antineoplastic actions. Moreover, thyme has long been known for its antiviral, antibacterial, antifungal, and antiseptic activities, in addition to remarkable disruption of microbial biofilms. In the COVID-19 era, some thyme constituents were investigated for their potential in viral binding. As such, thyme presents a wide range of functional possibilities in food, drugs, and other fields and prominent interest as a nutraceutical. The aims of the current review are to present botanical and nutritive values of this herb, elaborate its major constituents, and review available literature on its dietetic and biological activities.

## 1. Introduction

Wild edible plants (WEPs) as defined by the food and agricultural organization (FAO) are “the plants that grow spontaneously in self -maintaining populations in natural or semi-natural ecosystems and can exit independently of direct human actions” [1]. In fact, as summarized by Shumsky and Colleagues [2], WEPs are characterized by being locally available and known traditionally through generations, less expensive, having high advantages for poor populations, available during stressful conditions such as drought or famine periods and finally, being able to resist climate change. Moreover, WEPs have been demonstrated to have an important role in providing plenty of nutritional requirements that are important for improving health, thereby contributing to reduce food insecurity and scarcity, famine, or conflict [3,4]. Therefore, the trend towards implanting WEPs in food dishes and recipes nowadays, seems to have increased and become more popular than few years ago. According to a recent estimate, more than 8000 plant species are distributed all over the world, from these only 100 provide the majority of the world’s food. Among these 100, less than 20 species are used in food preparation [5]. China is characterized by having the oldest and biggest original centers for vegetables in the world, estimated to be around 213 families, 815 genera and 1822 species of plants. In North America, natives consume many WEPs daily; they constitute a good example of the ethnographical use of these natural sources. In Africa, Latin America, and the subcontinent of India, many WEPs are cultivated and consumed [5]. For example, a study was done in the city of Bingol in Turkey and showed that most of the consumers in this area eat these plants in their raw form; others use the flowers and branches for preparing herbal tea. In addition, many use them as spices and for commercial purposes [6].

Specifically, in the Mediterranean region, there are an estimated 25,000 to 30,000 species of WEPs, many of which are endemic to the region. The diversity of species in this region can rely on the ecological conditions, such as the convenient climate and the soil type [5]. In Lebanon, WEPs are estimated at about 2600 species (12% of total species in the Mediterranean region) and are mainly consumed within rural areas for their health and medicinal qualities, either raw without any preliminary preparation, or as snacks, providing important sources of nutrients absent in modern high-fat, high-sugar snacks [7]. In addition, several types of these plants are used in fresh salads. Most well-known cooking methods of such plants are simply fried with onions or used in omelet preparations [7,8]. A study was done in Lebanon by conducting semi-structured interviews in which ethno-pharmacological information was collected. As such, 53 native informants in 13 towns and villages surrounding Mount Hermon were asked about wild plants they use. The results showed that 124 plant species are still used by local communities in traditional medicine as a beneficial source for the treatment of different illnesses [9].

The *Lamiaceae* family is particularly well represented in Lebanon, where 136 species belonging to 29 genera have been inventoried. Some of them can be considered main ingredients in popular Lebanese dishes as spices such as *Origanum syriacum*, *Satureja thymbra*, *Thymbra spicata* and *Thymus vulgaris*. Others are eaten as salads such as *Salvia rosemarinus*, *Coridothymus capitatus*, and *Salvia fruticosa* which are also used in traditional medicine as a cure for different diseases, such as microbial infections [10]. A study done on eleven *Lamiaceae* species involving their chemical composition and the antimicrobial activity of their essential oils, revealed that *Lamiaceae* species are mostly used against gastrointestinal infections [10]. The beneficial effects of many plants and their use as pharmaceutical drugs are demonstrated by clinical and preclinical studies [11].

One of the most popular WEPs native to the Mediterranean region including Lebanon is thyme (*Thymus vulgaris*); it is one of the most important edible plants, having many benefits. Thyme is rich in phytonutrients, minerals, vitamins, flavonoids and antioxidants. In addition, the therapeutic effects of thyme and its essential oils, especially thymol and carvacrol, against various diseases were demonstrated in several studies. This is attributed to its multi-pharmacological properties that include, but are not limited to, antioxidant, anti-inflammatory, and antineoplastic actions. Moreover, thyme has long been known for its antiviral, antibacterial, antifungal, and antiseptic activities. The current review presents the history, botanical and morphological properties of thyme, together with its nutritive and chemical values, and reviews available literature on its major biological activities. Literature for this narrative review was collected by a search for published articles about thyme on PubMed and Google Scholar, to allow consideration of a large spectrum of available evidence on this herb. By surveying the current literature using the key words thyme, *Thymus vulgaris*, thyme essential oil, and thymol, authors were able to include pertinent studies and summarize their findings in a format accessible to the reader. Articles which do not specifically mention *Thymus vulgaris* or which do not relate to the physiological functions of this herb addressed in this review were excluded.

## 2. Thyme (*Thymus vulgaris*): An Overview

*Thymus vulgaris* commonly known as “thyme” has been used for many centuries for its flavoring, culinary, and medicinal properties [12]. The name thyme derives from the Greek word ‘thymos’ which means courage or strength. In the first century AD, thyme was used mainly as a medicinal plant, which was mentioned in Dioscorides’ work. However, in the Mediterranean region, it was used mainly as spice and then spread all over the world [12].

### 2.1. Systematic Classification and Distribution

The *thymus* genus is a group of aromatic plants, belonging to the *Lamiaceae* family (Labiate). According to Jalas [13] and Morales [14], 214 species and 36 sub-species are known and divided into eight sections: *Micantes*, *Mastichina*, *Piperella*, *Teucrioides*, *Pseudothymbra*, *Thymus*, *Hyphodromi* and *Serpyllum*. *T. vulgaris* L. and *T. zygis* L. belong to the Western Mediterranean area. *Thymus. T. vulgaris* is native to Southern Europe, from Spain to Italy [12]. In Lebanon, *T. vulgaris* is the native species known, according to Talhouk et al. [15]. Below, the systematic classification of this species is listed:

Kingdom: Plantae
Subkingdom: Tracheobionta
Superdivision: Spermatophyta
Division: Magnoliophyta
Class: Magnoliopsida
Subclass: Asteridae
Order: Lamiales
Family: Lamiaceae
Genus: *Thymus* L.
Species: *Thymus vulgaris* L. [16]

### 2.2. Thyme Botanical Aspects

**Figure 1:**
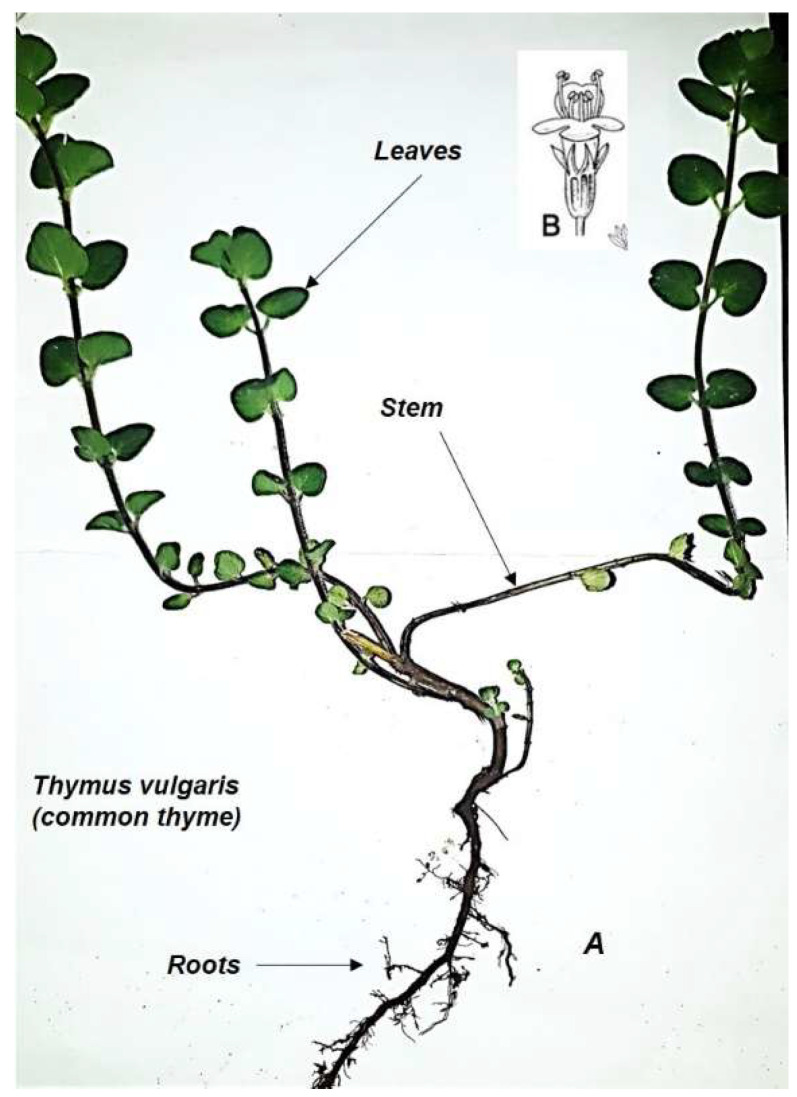
*Thymus vulgaris* (common thyme), sketch drawing, not necessarily to scale. (**A**) Plant vegetative parts: leaves, stem, and roots. (**B**) Reproductive part: flower of a cyme type, purple and white color, bisexual and two-lipped with a hairy glandular calyx responsible for a pleasant scent. The sketch is courtesy of the authors.

*T. vulgaris* is a perennial, evergreen subshrub with a generally upright, woody-based stem. The leaves are arranged as whorls around the stem, evergreen and simple [15]. They have an ovate shape, fine texture and a pleasant scent. They mainly constitute the edible part for humans. The flowers are of a cyme type, purple and white in color, bisexual and two-lipped with a hairy glandular calyx giving a pleasant scent. Their blooming occurs during spring and summer [12]. *T. vulgaris* has a moderate growth rate. At maturity, it reaches 0.5 to 1 m in height and spreads over 0.5 to 1 m and sometimes 15 m on the ground as a green cover. It takes 2 to 5 years to reach its maximum height [15]. It can tolerate frost and drought and poor, salty environments. It lives in loamy and sandy soils, having a neutral and alkaline pH. In Lebanon, *T. vulgaris* grows in groups on calcareous rocks. It does not need a lot of water but requires full sunlight. It has no invasive potential and can live for a maximum of 25 years [15]. *T. vulgaris* is most affected by root rot disease. Finally, thyme can be propagated from seeds, cuttings or by layering [15]. Figure 1 represents a botanical sketch of thyme.

## 3. Chemical Composition and Essential Oils of Thyme

Many studies have been conducted on thyme species to identify their chemical composition. A wide variety of chemical compounds as well as essential oils constitute the main composition of thyme that varies with climate and geographical area. Investigations have reported that thyme contains 56.53% monoterpenes, 28.69% monoterpene hydrocarbons, 5.04% sesquiterpene hydrocarbons and 1.84% oxygenated sesquiterpenes [17]. Thyme is rich in many flavonoids and phenolic antioxidants like zeaxanthin, lutein, pigenin, naringenin, luteolin and thymonin [16]. Fresh thyme has one of the highest antioxidant levels being rich in minerals and vitamins that are essential for optimum health. Potassium, iron, calcium, manganese, magnesium and selenium are concentrated in the leaves, and the main constituent of the oil extracted is thymol. Thymol is an important phenolic component mainly responsible for thyme’s antioxidant activity [16]. The thyme’s flowered stem contains flavonoid derivatives such as apigenol and luteolol, phenolic acids such as cafeic and rosmarinic acids, and tannins [18].

**Figure 2:**
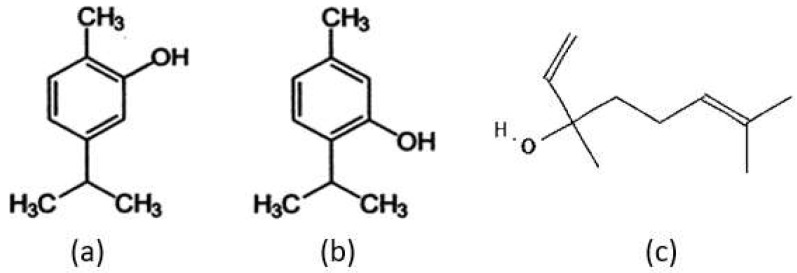
The chemical structure of thymol (**a**), carvacrol (**b**), and (**c**), linalool [16]. Linalool structure was retrieved from data deposited in or computed by PubChem (https://pubchem.ncbi.nlm.nih.gov, accessed on 1 January 2020).

To determine the fractions of essential oils in the thyme herb, standardized and appropriate techniques are always recommended. Extraction followed by analytical characterization are the main steps followed to build the profile of essential oils in thyme. Using capillary electrochromatography coupled to diode array detection (CEC-DAD) and liquid chromatography-tandem mass spectrometry (LC-MS/MS), thymol and carvacrol (Figure 2) were determined as the main constituents of thyme [19]. On the other hand, using gas chromatography-mass spectrometry (GC-MS) and high performance liquid chromatography-ultraviolet (HPLC-UV), it was found that thyme contains 48.19% linalool and 21.3% carvacrol as the major terpenes [20]. Despite the technique used to identify the different fractions of essential oils in thyme, some components are always encountered in all species at variable amounts. This diversity is due to climate, soil, harvest period and the method of preservation [18]. The major classes of chemical compounds found in thyme are monoterpenes, bicyclic monoterpenes, monoterpenols, and bicyclic monoterpenols as well as sesquiterpene lactones [19].

Essential oils are natural mixtures of a large variety of components at different concentrations. *T. vulgaris* contains at least six chemotypes of essential oils: thymol as the major constituent, carvacrol, p-cymene, ɤ-terpinene, linalool, β-myrcene and terpinen-4-ol as well as others referred to in Table 1 [16].

## 4. Thyme Nutritional Value and Health Benefits

Thyme is loaded with phytonutrients, minerals and vitamins that are vital for good health. These nutrients are known for their disease-preventing and health-promoting properties and contribute to the benefits of this herb. Thyme is particularly rich in vitamin A and vitamin C. Vitamin A is an antioxidant known for being vital in maintaining healthy mucus membranes and skin as well as good vision. Vitamin C is essential to resist infectious diseases and protects against harmful pro-inflammatory free radicals. Thyme also contains B-complex vitamins, mainly vitamin B6 (pyridoxine), that assists in maintaining the γ-aminobutyric acid (GABA) levels in the brain and acts as a stress reliever. Vitamin K, vitamin E and folic acid are also present in thyme [16]. According to the US Department of Agriculture National Nutrient database, the oxygen radical absorbance capacity (ORAC) value of thyme is 27,426 µmoles of Trolox Equivalents per 100 g (molTE/100 g) [21]. This value indicates the power and capability of an antioxidant product to neutralize the free radicals.

Thyme is also loaded with minerals that are essential for good health. Its leaves form an excellent source of potassium, calcium, iron, manganese, magnesium and selenium. Potassium is an important component of cells and body fluids and controls heart rate and blood pressure. Iron is crucial in red blood cell formation, and manganese is a co-factor for the antioxidant enzyme, superoxide dismutase [16]. A summary of the major nutritive components of thyme is shown in Table 2 [16].

## 5. Applications and Uses of Thyme in Food Industry

The food industry, one of the largest industries across the globe, has encountered many changes throughout the last decades and shifted towards healthier applications and innovative products. The plant extract industry infiltrates the food industry since plants have been used historically as food preservatives. Plants contain phytochemicals that protect them from microbial contamination and spoilage.

Plant extracts such as essential oils, have been used to preserve food, increase its antimicrobial activity and improve its organoleptic properties. Thymol, the main essential oil constituent in thyme, is well known for its antioxidant, anti-inflammatory and antimicrobial activity. Its usage in food, as well as dried thyme leaves, is limited almost entirely to the meat industry and as condiment to replace or decrease the use of unhealthy synthetic additives [22].

When thyme is added to food, some features may be improved, such as storage conditions, composition, and antimicrobial activity due to its preservative properties. Moreover, the presence of antioxidants and micronutrients in thyme can reduce the bioactivity of the food commodity. The only limiting aspect of thyme addition in some foods is the development of a negative organoleptic effect that sometimes contributes to an unpleasant odor and taste [22]. To counter these problems, essential oils are encapsulated with nanocarriers such as nanofibers, cyclodextrins or amylose, which mask the flavor and increase the stability of the volatile compounds in essential oils. As another strategy, thyme essential oils are sometimes combined with other antimicrobial and antioxidant compounds to provide synergetic effects, thus decreasing the negative organoleptic aspect [22].

## 6. Biological Activity of Thyme

**Figure 3:**
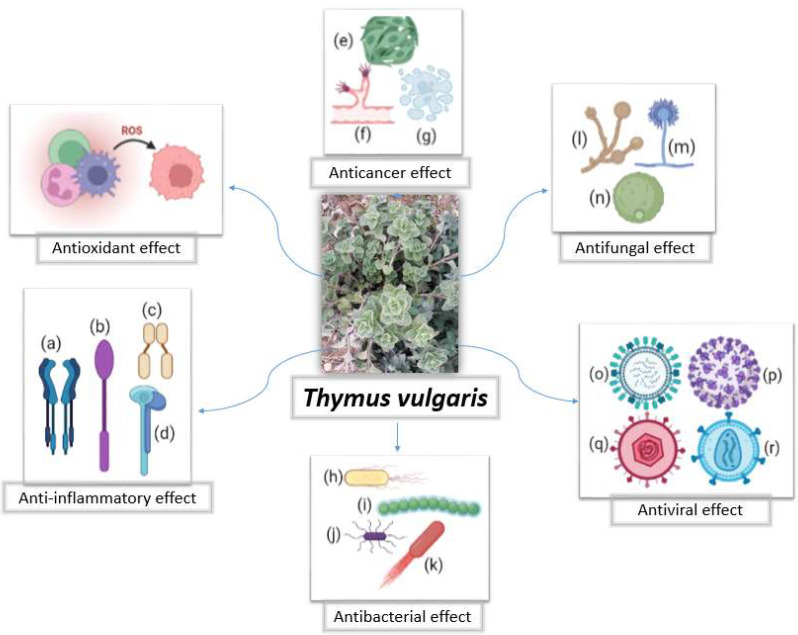
A photo of *Thymus vulgaris* with a summary of some of its biological effects. ROS: reactive oxygen species; (**a**) interferon; (**b**) tumor necrosis factor (TNF)-α; (**c**) nuclear factor-kappa B (NF-кB); (**d**) interleukin; (**e**) proliferation; (**f**) angiogenesis; (**g**) apoptosis; (**h**) *Escherichia coli*; (**i**) *Streptococcus*; (**j**) *Bacillus*; (**k**) *Listeria*; (**l**) *Candida*; (**m**) *Aspergillus*; (**n**) *Cryptococcus*; (**o**) influenza virus; (**p**) severe acute respiratory syndrome coronavirus-2 (SARS-CoV-2); (**q**) herpes virus; (**r**) human immunodeficiency virus (HIV). Thyme photo is courtesy of the authors and the figure was prepared using BioRender.com, accessed on 31 March 2022.

A review of major biological and therapeutic effects of thyme and its main constituents is presented below, with a focus on antioxidant, anti-inflammatory, anticancer, and antimicrobial properties. These properties are also represented in Figure 3.

### 6.1. Antioxidant Activity

Oxidation is a chemical process during which electrons or hydrogen are transferred from a certain substance to an oxidizing agent [23]. Besides, lipid oxidation is one of the major problems encountered during food processing, storage and consumption. Such reactions result in the formation of reactive oxygen species (ROS) and free radicals that begin chain reactions [24]. This, in turn, leads to deterioration in food quality and stability, as well as, carcinogenesis, mutagenesis, inflammation, DNA changes, aging, and cardiovascular diseases [25,26,27]. Antioxidants are molecules that stop the initiation or propagation of oxidizing chain reactions, thereby, delaying or inhibiting the oxidation of lipids or other molecules [28].

In general, the antioxidant properties possessed by thyme extracts is primarily due to their richness in phenolic compounds and is related to their ability to act as free radical scavengers, metal ion chelators and inhibitors of oxidative enzymes [29]. Several in vitro and in vivo methods have illustrated the antioxidant activity of thyme. For instance, Wisam et al. [30] revealed the antioxidant activity of *T. vulgaris* and its reducing power that might be attributed to higher amounts of total phenols and flavonoids. It was explained that the phenolic compounds possessed antioxidant effects mainly due to their redox properties, which can play a significant role in adsorbing and neutralizing free radicals, quenching singlet and triplet oxygen, or decomposing peroxides.

Moreover, Lee and Shibamoto conducted a study to determine the antioxidant potential of volatile extracts isolated from various herbs and spices, including thyme, which produced better results, with an inhibitory effect similar to that of α-tocopherol or butylhydroxytoluene [31]. Furthermore, Tohidi et al. [32] investigated the antioxidant activity of *Thymus* species collected from different regions of Iran using 1,1-diphenyl-2-picrylhydrazyl (DPPH) and a reducing power assay. It was found that *T. vulgaris* was among the thymus species that possessed higher antioxidant activities than the others. Also, in a study performed by Roby et al. [33], thyme methanol extract possessed the best antioxidative activity, which was better than those of other plants (sage and marjoram), α-tocopherol and the synthetic antioxidant butylated hydroxy anisole (BHA).

Findings of a study conducted by Zaborowska et al. showed that the stability of sunflower oil was prolonged by ethanol thyme extract that might be a potent antioxidant for its stabilization [24]. Furthermore, El-Guendouz et al. also evaluated the antioxidant activity of thyme waste extract in oil in water (O/W) emulsions. It was found that the extract had the capacity of preventing the formation of primary and secondary lipid oxidation products in O/W emulsions, constituted by diverse proportions of wheat and almond oils, without interfering with the viscosity parameters, for 10 weeks, at 37 °C. The combination of higher concentrations of thyme waste extract (0.02%, 0.04%) and almond oil (≥50%) were the best in protecting the primary oxidation of emulsion samples [34]. Moreover, an experiment was performed by El-Nekeety et al. to determine the antioxidant properties of *T. vulgaris* oil against aflatoxin-induced oxidative stress in male rats. The results indicated that the combined treatment with aflatoxins and the oil showed significant enhancements among all tested parameters that were more pronounced within the group which received the oil at a high dose [35].

The in vitro antioxidant activity of thymol, a major polyphenolic compound in thyme, was investigated by Yu et al. [36]. Results showed that thymol exhibited antioxidant activity and may suppress the progression of high-fat-diet-induced hyperlipidemia and atherosclerosis through decreasing aortic intimal lipid lesions, reducing oxidative stress and serum lipids. Besides, a study conducted by Nagoor Meeran and Prince [37] confirmed thymol’s potent antioxidant action through enhancing the activity of endogenous antioxidant enzymes, such as superoxide dismutase, catalase, glutathione peroxidase, glutathione-S-transferase, and the level of other non-enzymatic antioxidants such as vitamin C, vitamin E, and reduced glutathione, and therefore significantly increasing the total antioxidant status in vivo [38].

Additionally, the efficacy of thymol and carvacrol as antioxidants in microencapsulated walnut oil was recently evaluated by Gursul et al. [39]. It was suggested that walnut oxidation was reduced by fortification with thymol and carvacrol, and encapsulation led to improving the storability, providing extensive interaction and stabilizing lipid radicals. Lukic et al. [40] had also estimated the antioxidant activity of films impregnated with thymol (27.5%), carvacrol (21.2%) and their combination (21.5%). The best antioxidant activity, measured using a DPPH assay, was illustrated by the film impregnated with the mixture of thymol and carvacrol, attributed to their synergistic interactions and reducing power, with good storage stability.

The effects of thymol and carvacrol on sperm quality oxidative stress and the antioxidant system in rats was investigated by Güvenç et al., whose findings demonstrated that these compounds decreased oxidative damage and improved sperm quality [41]. Furthermore, thymol essential oils were found to be useful in lowering lipid peroxidation in fish (Nile tilapia) meat, increasing antioxidant enzyme activity and having a positive effect on their growth [42,43]. In addition, they increased the stability and improved the quality of minced pork by retarding its lipid oxidation [44].

### 6.2. Anti-Inflammatory Activity

Inflammation is a complex, natural, protective response of body tissues as a defense mechanism against harmful stimuli, including pathogens and cellular injury [45,46]. The use of thyme and its extracts has been traditionally practiced around the globe for the treatment of inflammatory diseases [46,47], and various studies have presented its anti-inflammatory properties. In this context, the effect of thyme extracts was evaluated on oxidized-LDL-activated THP-1 macrophages by measuring the expression and release of some inflammatory mediators. It was demonstrated that thyme extracts exerted a dose-dependent decrease in the production and gene expression of the proinflammatory mediators tumor necrosis factor (TNF)-α, IL-1B, and IL-6 associated with an increase in the anti-inflammatory IL-10 cytokine secretion in activated macrophages [48]. Moreover, Habashy et al. [49] examined the anti-inflammatory properties of Greek *T. vulgaris* oil and water extracts and their ability to detoxify the lipopolysaccharide (LPS)-induced inflammation and oxidative stress. According to their findings, these extracts were able to reduce the LPS-induced elevation in cyclooxygenase (COX)-2, nuclear factor-kappa B (NF-кB), inducible nitric oxide (NO) synthase (iNOS), TNF-α, and NO, and produced a more potent attenuating effect than dexamethasone for most of the studied inflammatory mediators.

Golbahari et al. [50] conducted a study on 50 rats with rheumatoid arthritis to evaluate the anti-inflammatory effects of thymol (100 mg/kg orally) or nicotine (2.5 mg/kg orally) alone or in combination. Obtained findings showed that each of thymol and nicotine reduced TNF-α, IL-6, IFN-, IL-1β and IL-17 levels, however, a thymol and nicotine combination (50 and 1.25 mg/kg, respectively) produced greater reduction in IL-1β, IL-17, C-reactive protein and myeloperoxidase. In another study, the anti-inflammatory potential of thyme essential oil was screened by evaluating the exudate accumulation in the pleural cavity and leukocyte migration after carrageenan injection in mice. It was demonstrated that thyme essential oil significantly reduced inflammatory exudates at doses of 250, 500, and 750 mg/kg and reduced the number of migrating cells at a dose of 750 mg/kg. On the other hand, groups treated with indomethacin and celecoxib revealed a decrease in inflammatory exudates but not in leukocyte migration [51]. Besides, in vivo anti-inflammatory activities of *T. vulgaris* essential oils were proven by significantly reducing carrageenan-induced paw edema in mice [52]. Furthermore, unfractionated essential oil from *T. vulgaris* was found to reduce neutrophil infiltration during an inflammatory response in zebrafish [45].

A study has also assessed the anti-inflammatory potential of *T. vulgaris* aqueous extract by evaluating its effect on NO production induced by LPS and interferon-γ (IFN-γ) in the murine macrophage cell line J774A.1, and by evaluating the scavenging activity and inducible nitric oxide synthase (iNOS) mRNA expression. Findings revealed a significant dose-dependent inhibition in NO production that is induced by LPS and IFN-γ, scavenging of NO radicals released by an NO donor, PAPA-NONOate, and inhibition of iNOS mRNA expression [53]. Another study performed on human macrophage-like U937 cells showed that carvacrol, as a component of thyme oil, possesses anti-inflammatory properties by suppressing LPS-induced COX-2 mRNA and protein expression through its agonistic effect on PPARgamma [54].

### 6.3. Antineoplastic Activity

Cancer is considered a serious threat globally [55]. It ranks as a leading cause of mortality worldwide, accounting for nearly ten million deaths in 2020 and is a growing threat with the incidence expected to increase 47 percent by 2040 [56,57]. The chemotherapeutic antitumor drugs used traditionally for the treatment of cancer patients showed high cytotoxicity to the tumor as well as normal tissues [55,58]. Therefore, searches for new therapeutic options, including medicinal plants and their phytochemicals, has been promoted due to their lower risk of side effects compared to standard chemotherapeutic drugs [59]. Numerous studies have demonstrated the beneficial effects of different medicinal plant extracts and their ability to exert damaging effects on cancer cells through various molecular mechanisms, including intrinsic or extrinsic caspase-dependent apoptosis, autophagy, and necroptosis [60,61,62].

Thyme possesses numerous compounds, especially the monoterpenoid phenols carvacrol and thymol, which have great potential to be used in therapeutic and management interventions against cancer due to their pharmacological properties [27,63,64]. Carvacrol and thymol exerted anticancer effects in various types of cell lines mimicking human cancers and they demonstrated their potential as chemopreventive or anticancer agents in different types of cancers [55,65,66]. Their major mechanisms of anticancer actions include induction of apoptosis, inhibition of cell growth (antiproliferative effect), augmentation of ROS generation, depolarization of mitochondrial membrane, activation of Bax proapoptotic mitochondrial proteins, inhibition of angiogenesis, interaction with caspase or poly-ADP ribose polymerase, and diminution of tumorigenesis by modulating the activity of carcinogen metabolizing enzymes [59,65,67].

The antitumor effects of *T. vulgaris* were evaluated in vivo and in vitro in mammary carcinoma models where two concentrations (0.1% and 1%) of dried *T. vulgaris* were continuously administered in the diet of a chemically-induced rat mammary carcinomas model and a syngeneic 4T1 mouse model. It was found that *T. vulgaris* at both doses reduced the volume of 4T1 tumors by 85% (0.1%) and 84% (1%) in mice compared to the control and the treated tumors showed a substantial decrease in necrosis/tumor area ratio and mitotic activity index. Furthermore, carcinoma cells showed a decrease in CD44, VEGFR-2, and ALDH1A1 expression, a decrease in malondialdehyde (MDA) levels, a decrease in the lysine methylation status of H3K4me3, and an increase in Bax expression. This showed that *T. vulgaris* has significant chemopreventive and therapeutic activities against experimental breast carcinoma [68]. Additionally, *T. vulgaris* extracts were reported to inhibit the proliferation of colorectal HCT116 cancer cell lines, increase their apoptotic cell death, and reduce their adhesion to fibronectin [69]. The antiproliferative activity of *T. vulgaris* L. essential oil was also analyzed against the breast adenocarcinoma (MCF-7) cell line, lung carcinoma (H460) cell line and acute lymphoblastic leukemia (MOLT-4) cell line, revealing a dose-dependent inhibition of cell proliferation in all tested tumor cell lines with different sensitivities [70]. Thyme essential oil was also able to inhibit the cell growth of human head and neck squamous cell carcinoma (HNSCC) through regulating interferon signaling, N-glycan biosynthesis and ERK5 signaling [71].

Thymol demonstrated proapoptotic and antiproliferative properties in lung, breast and prostate cancer cell lines, hence, it could serve as a potential therapeutic agent in the future [55]. A study was conducted by Günes-Bayir et al. [72] to evaluate and compare the impact of various thymol doses on human fibroblast and gastric adenocarcinoma cells. Findings revealed that thymol at low concentrations provided antioxidative protection to healthy cells in vitro, while inducing toxic effects in cancerous cells at all thymol concentrations. In addition, Aydan et al. [73] confirmed that thymol may possess antiproliferative potential against brain tumor cells where it was able to reduce the cell viability in cultured primary rat neurons at a concentration of 400 mg/L and to inhibit cell growth in N2a cells at concentrations of 200 and 400 mg/L. Moreover, thymol administration (20 mg/kg/day, p. o.) to male Wistar rats showed promising protective effects against colon cancer by significantly reducing elevated serum levels of colon-related tumor markers, carbohydrate antigen 19-9 (CA 19-9) and carcinoembryonic antigen (CEA), as well as the apoptotic marker, caspase-3 compared to the colon cancer group [74]. Li et al. [75] had also investigated the efficacy of thymol in bladder cancer cells revealing that it inhibited cell proliferation in a dose and time-dependent manner. Thymol induced cell cycle arrest at the G2/M phase, generation of ROS, and apoptosis through the intrinsic pathway, caspase-3/9, JNK, and p38 activation, release of cytochrome C and down-regulation of anti-apoptotic Bcl-2 family proteins.

Several studies have demonstrated that carvacrol exhibits strong antitumor effects and it was shown to be more cytotoxic compared to thymol [18,76]. In relation to breast cancer, treatment with carvacrol reduced the viability of MDA-MB231 and MCF-7 cells lines [76,77] by regulating the cell cycle with the TRPM7 pathway being one of the pharmacological mechanisms [77]. Also, carvacrol at a concentration of 10–600 μmol/L significantly reduced the cell viability of gastric adenocarcinoma (AGS) in a dose-dependent manner by exerting cytotoxic, genotoxic, apoptotic, ROS generating, and GSH-reducing effects on AGS cells [78]. With respect to colon cancer, carvacrol inhibited the proliferation and migration of two human colon cancer cell lines, HCT116 and LoVo. It was able to reduce the expression of metalloproteinase-2 and -9 (MMP-2 and MMP-9), Bcl-2, cyclin B1, p-ERK, and p-Akt levels, and to increase p-JNK and Bax levels, resulting in cell cycle arrest at the G_2_/M phase [79]. Furthermore, Jung et al. [80] demonstrated that carvacrol can inhibit cell proliferation and migration in non-small cell lung cancer (NSCLC) by down-regulating tyrosine kinase receptor (AXL) expression and inhibiting the phosphorylation of AXL upon ligand stimulation. Khan et al. [81] examined the anticancer mechanism of carvacrol against human prostate cancer cells, where it was revealed that it exhibited antiproliferative action against DU145 cells in a concentration and time dependent manner. Cell cycle arrest at the G_0_/G_1_ phase, augmentation of ROS generation and disruption of the mitochondrial membrane potential were induced, in addition to apoptosis, confirmed by activation of caspase-3.

### 6.4. Antibacterial Activity

Like other plants, and due to biological and structural diversity of its components, thyme may be considered a renewable source for diverse antibacterial compounds, with *T. vulgaris* being the most investigated species [82]. Globally, antimicrobial resistance (AMR) poses a serious threat to human health. According to recently published predictive statistical models and comprehensive assessments, AMR is a leading cause of death around the world, with the highest burden in low-resource settings. Approximately five million deaths were associated with bacterial AMR in 2019 alone. The six leading pathogens for resistance-associated deaths were *Escherichia coli*, *Staphylococcus aureus*, *Klebsiella pneumoniae*, *Streptococcus pneumoniae*, *Acinetobacter baumannii*, and *Pseudomonas aeruginosa* [83]. Several existing gaps complicate the problem of AMR, including extremely slow innovation, vaccine shortages, dry clinical pipelines, and episodic, uneven action from policymakers [84]. Data show that AMR growth rates exceed all efforts to ameliorate the situation, and the search for new antibiotic classes remains one of the most important strategies in the battle against this crisis [85]. In the search for creative approaches to tackle AMR, natural products offer a promising supply of antibacterial lead compounds, which could help fill the drug discovery void as the antibiotic resistance situation worsens [86]. As such, and at least from known use in traditional medicine, thyme possesses significant antimicrobial effect on several human, animal and plant pathogens, advocating for further research into this herb, particularly with clinical trials and realistic dosages [82].

The antiseptic qualities of thyme as an aromatic and medicinal plant have been recognized since antiquity, and it has been used to treat malaria since the sixteenth century. *The British Herbal Pharmacopoeia* classifies thyme as a medicinal plant, and among the indications for its use, it mentions bronchitis, bronchial catarrh, whooping cough, and sore throat, and recommendations are given for combining it with other plants. Attempts to characterize the antimicrobial properties of thyme in the laboratory date back to the early 1900s [87]. The essential oil of thyme has been recognized to exhibit significant antibacterial activity [88,89] associated with the phenolic components, carvacrol and thymol, and many studies have elaborated this property. For instance, in 2014, Borugă and Colleagues assessed the antibacterial effects of essential oil of *T. vulgaris*, where reference strains of *S. aureus* ATCC 25923, *K. pneumoniae* ATCC 13882, *Salmonella typhimurium* ATCC 14028, *P. aeruginosa* ATCC 27853, *E. coli* ATCC 25922, and *Enterococcus faecalis* ATCC 29212 were exposed to different concentrations of the essential oil. The study revealed that thyme essential oil was mostly effective against *S. aureus* and *K. pneumoniae*, while its potency against other strains was dose dependent. The results demonstrated that the essential oil possesses strong antimicrobial properties, and may designate a new birthplace of natural antiseptics with uses in both the pharmaceutical and food industry [90].

Burt and Reinders [91] examined the effect of thyme essential oil on the enteric pathogen *E. coli* O157:H7, which remains an important contaminant in food production, and should be efficiently eliminated from meat, milk, water, vegetables, fruits and fruit juices. Thyme essential oil was shown to possess significant in vitro colicidal and colistatic activity over a broad temperature range, and this was substantially improved by the addition of agar as stabilizer in the testing medium. These findings indicated that thyme essential oil could be further examined for application in the food industry to improve food safety. These original findings were recently augmented by the use of thyme oil nanoemulsions aided by ultrasound to decontaminate the surface of vegetables against *E. coli* O157:H7 [92]. Moreover, in another investigation, thyme essential oil exhibited a synergistic effect with enterocin A, a small, natural antimicrobial bacteriocin, on *E. coli* O157:H7. Treatment with enterocin A alone did not affect the growth of *E. coli* O157:H7. However, the combination of thyme essential oils and enterocin A yielded a synergistic antimicrobial effect and a decrease in the minimum inhibitory concentration (MIC) of the essential oil. The combination exhibited enhanced bactericidal effect against *Listeria monocytogenes*, a foodborne pathogen. These data indicate that the combination may be exploited as a suitable control strategy for *E.coli* O157:H7 and *L. monocytogenes*, considering both economical aspects and the food flavor, and holding the additional promise of minimizing the development of resistance [93]. Furthermore, in systems of beef and cheese, thyme oil showed protective effect against *E. coli* O157:H7 and vancomycin-resistant enterococci, adding to the repertoire of food contaminants towards which this oil shows antibacterial activity [94,95]. In an investigation of 15 different plant oils against several food-borne pathogenic bacteria (*L. monocytogenes*, *S. typhimurium*, and enterohemorrhagic *E. coli* O157:H7) and food spoilage bacteria (*Brochothrix thermosphacta* and *Pseudomonas fluorescens*), thyme essential oil was tested. The essential oils of cinnamon, oregano, and thyme had the strongest antibacterial activity attributed to the key bioactive constituents, namely cinnamaldehyde, carvacrol, and thymol. This suggests thyme essential oil as a further alternative for food safety whose efficiency warrants further evaluation [96].

Apart from foodborne pathogens, and as far as clinical strains are concerned, data on the activity of thyme against multidrug resistant clinical bacteria are accumulating. Almost a decade ago Sienkiewicz et al. explored the antimicrobial activity of thyme essential oil against clinical multidrug resistant *Staphylococcus*, *Enterococcus*, *Escherichia*, and *Pseudomonas* genera, that were isolated in the hospital setting from infections of the oral cavity, abdominal cavity, respiratory tract, genitourinary tract, and skin. Using an agar diffusion method, the essential oil of thyme was shown to strongly inhibit the growth of the tested clinical strains, suggesting that it may be reasonable to investigate it as a phytopharmaceutical for treatment and prevention of bacterial infections caused by both Gram-positive and Gram-negative bacteria [97]. The results were reproducible with a panel of 30 *E. coli* strains isolated from patients with various conditions, where thyme essential oil was active against all the strains and was more potent than other oils [98]. In a recent investigation on nosocomial *A. baumannii* and *K. pneumoniae*, thyme essential oil decreased the MIC of the antibiotic colistin by 8- to 64-fold and 8- to 128-fold in colistin-resistant and colistin-susceptible strains. Hence, the essential oil of thyme can improve the efficacy of colistin and significantly reduce its concentration needed to inhibit both bacteria, and can be tested as a promising antimicrobial adjuvant [99]. Loose et al. tested the activity of thyme essential oil on uropathogenic bacteria obtained from clinical isolates and detected a synergistic activity of combined thyme and tea tree oil. The combination increased the activity of fosfomycin and pivmecillinam, but not nitrofurantoin, against *E. coli*. This research laid the groundwork for further study into the potential of thyme as an alternate or supplemental treatment for urinary tract infections [100].

Regarding the effect of thyme on *Streptococcus* species, data have been documented for *S. mutans,* that causes dental caries but might also cause infective endocarditis [101], *S. pyogenes* that causes sore throat or pharyngitis [102], and the zoonotic pathogen, *S. suis* [103,104]. Interestingly, a novel model of delivery of topical products for acne vulgaris, an antimicrobial, standardized, nano-emulgel was synthesized from thyme and clove essential oils, and screened in vitro and in vivo against clinical skin bacterial isolates (*Pseudomonas stutzeri*, *Enterococcus faecium* and *Bacillus thuringiensis*). The nano-emulgel revealed bacteriostatic and biofilm inhibition properties, and improved skin histological structures in rat models, indicating potential for a bacteriostatic action and non-antibiotic microbial pathway inhibition as a topical clinical alternative to available agents [105]. Prepared as a microemulsion, and recently in 2022, thyme oil proved to be a successful alternative economic choice for multidrug resistant *Salmonella* Enteritidis treatment in poultry farms, decreasing the fecal content and mortality rate due to this pathogen [106].

Despite such promising data, challenges remain for the proper incorporation of thyme and its oil as agents directed towards infection. These include low water solubility, sensitivity to light, moisture, heat, and oxygen, and various chemical and structural instabilities, in addition to the need for clinical data. Recent efforts for the nanoliposomal formulation of thyme essential oil and its encapsulation in chitosan nanoparticles are being attempted and may represent a valid alternative to circumvent some of these challenges in the interest of thyme oil use for antibacterial and food safety purposes [107,108].

### 6.5. Antifungal Activity

The antifungal activity of thyme is mainly contributed to the phenolic compounds, thymol and carvacrol, where the latter was reported more than two decades ago, to have potent fungitoxic activity when tested on fruits to inhibit the growth of fungi [109]. Regarding the antifungal capacity, findings of the different studies are not always comparable, due to variances in herb quality, qualitative and quantitative variations in essential oil constituents, differences between fungal strains examined, and methodological differences [110].

Among the most common pathogens in fungal infections is *Candida*, some species of which are opportunistic pathogens and cause infections in immunocompromised or otherwise impaired hosts. Infections may be superficial or invasive, with the latter possibly life-threatening. Azoles and echinocandins are antifungal drugs used globally to treat *Candida* infections. However, resistance to these antifungal drugs has increased in many species, adversely affecting clinical settings and patient treatment [111]. As such, and with the known bioactivity of thyme and its potential antifungal properties, studies evaluating the herb’s effect on *Candida* and its potential as a source of anti-candidal agents form a large body of literature. For example, the essential oil of thyme was studied against fluconazole-resistant isolates of *C. albicans* isolated from the blood or vagina. It successfully reduced the growth of the isolates on culture media and was fungistatic and fungicidal at low doses. The oil also inhibited germ tube formation and budding of fungal pathogens, and was more effective against resistant isolates than fluconazole [112]. In fact, the ability of *C. albicans* to change it morphology and form biofilms is central to its pathogenesis, as well to augmenting its resistance to antifungal agents [113]. As such, two independent research groups have investigated the effect of thyme essential oil on biofilm formation by *Candida*. In one study, thymol caused disaggregation and deformed shape of *C. albicans* biofilm cells and reduced hyphae formation in *C. tropicalis* biofilm. Also, thymol showed synergy with fluconazole against both the planktonic and biofilm modes of growth in both species [114]. In another study, thyme oil resulted in a statistically significant decrease in biofilm cell numbers, and diverse compounds from the oil were located in *C. albicans* cell wall, cell membrane, cytoplasm, and vacuoles, illustrating a multidirectional action together with the anti-biofilm activity [115]. Recently, both the oil of thyme and thymol at concentration below 16 mg/L were listed in a review of plant-derived preparations and compounds that inhibit *Candida* biofilm formation by at least 50% [116]. Furthermore, Rajkowska and Colleagues [117] showed that thyme oil induced large scale loss or decrease in enzymatic activity of *C. albicans*, along with changes in cellular and colonial morphology and in metabolic pathways like loss of the ability to assimilate saccharides. It was anticipated that such changes have a significant impact on *C. albicans* ability to cause infection. Apart from *C. albicans*, and considering non-*albicans* species and rare yeast species increasingly emerging as major opportunistic pathogens, thymol inhibited the growth of uncommon yeasts in vitro, suggesting it as a possible natural adjuvant for infections caused by those organisms [118].

Besides *Candida*, many species of the fungal genus *Aspergillus* can harm the health of plants, animals, and humans by direct infection and/or the formation of harmful secondary metabolites known as aflatoxins, which are among the most noxious mycotoxins [119]. A recent study by Oliveira and Colleagues [120] investigated the antifungal and anti-aflatoxigenic effects of thyme essential oil on *Aspergillus flavus*. Thyme essential oil exhibited antifungal activity through apoptosis, nuclear condensation, and plasma membrane damage. Additionally, the oil decreased aflatoxin production and gene expression and adversely affected secondary metabolism and mechanisms of virulence. In the assessment of 25 different plant essential oils against indoor species of *Aspergillus*, a study by Helbová et al. proved that thyme oil, combined with lemongrass oil, was the most potent synergistic antifungal against *A. fumigatus*. The synergistic combination may be useful for control of fungal growth or decreasing resistance to available synthetic antifungals [121]. Thymol, is approximately three-fold the inhibitory potential compared to thyme essential oil, and was capable of producing long-lived suppressive activity on different mold genera including *Aspergillus*, *Penicillium*, *Ulocladium*, *Absidia*, *Mucor*, *Cladosporium*, *Trichoderma*, *Rhizopus*, and *Chaetomium*, isolated from wall scrapings of damp dwellings in Croatia. This indicated potential for use of thymol or thyme essential oil at low concentration for disinfection of moldy walls [122]. Thyme proved to be a natural and cost-effective adjuvant when used in combination with itraconazole against *Cryptococcus neoformans* [123], a fungal pathogen that causes pneumonia and meningitis in immunocompromised individuals. In 2021, researchers from Korea investigated thymol mechanisms in the inhibition of *C. neoformans*. Among other pathways, thymol was found to regulate multiple signaling of calcineurin, and to reduce endogenous ergosterol content by decreasing the expression of ergosterol biosynthesis genes [124]. In terms of skin infection with dermatophytes that cause tinea, the essential oil of thyme showed interesting results when tested against *Microsporum* and *Trichophyton*, primary genera of dermatophytes obtained from clinical specimens, envisioning a natural alternative for topical antifungal drugs [125]. A nano-emulsion of thyme oil prepared by ultrasonification was among novel preparations that recently showed excellent fungicidal activity, not only against dermatophytes, but also against other molds and several pathogenic fungi of plants [126], perhaps highlighting a wealth of natural ingredients and a flexibility of design and formulation.

### 6.6. Antiviral Activity and Novel Findings in COVID-19

Among other extracts from the family *Lamiaceae*, the aqueous extract of thyme was tested against herpes simplex viruses type 1 and type 2 (HSV-1 and HSV-2) in a German study. The extract was able to inhibit both types of viruses in cell lines, affecting the viruses prior to being adsorbed on the surface of cells but not affecting intracellular viral replication. This shed a light on the prospective of these extracts for topical therapeutic application against recurrent herpes infections [127]. The results were replicated recently by Toujani and Colleagues [128], who demonstrated that ethanol extract had the strongest antiviral activity by direct inactivation of extracellular HSV-2 virions and, consequently, diminished ability of spread to new cells. With rhinoviruses and influenza viruses, responsible for the majority of acute viral respiratory infections, thyme was also investigated as an antiviral. In one study involving both viruses, thyme extract did not show anti-rhinovirus activity, but showed antiviral activity against the influenza virus. At non-cytotoxic concentrations, the extract reduced the cytopathic effect of influenza in a dose-dependent manner [129]. Some studies also showed promising anti-retroviral properties of thyme [130,131], suggesting it to possibly interfere with essential viral functions of HIV-1.

Recently, and with the coronavirus disease, COVID-19, perplexing healthcare systems and societies, intensive efforts to develop effective preparations against severe acute respiratory syndrome coronavirus-2 (SARS-CoV-2) were prominent, and natural compounds were not an exception. In this regard, the United States Environmental Protection Agency (EPA) placed thymol on the list of disinfectants with evidence for use against COVID-19, for disinfection of external hard surfaces and hands in healthcare, institutional, or residential applications [132]. Thyme essential oil has previously shown effectiveness against several RNA viruses including human and feline coronaviruses. This created outlooks for future applications and therapeutic possibilities for coronaviruses and modeling feline infection for the study of antivirals against human coronaviruses [133]. Javed and Colleagues [134] reviewed the biological and pharmacological properties of carvacrol within the scope of COVID-19. The potent antioxidant and immunomodulatory effects of this compound are thought to enhance host cellular immunity and interfere with ACE2 receptors, therefore may block the host cell entry of SARS-CoV-2. In addition, carvacrol interacts with viral protease and inhibits binding of the viral spike (S) glycoprotein to the host cell. In a computational evaluation study on plant essential oils, thymol was docked against the S1 receptor binding domain S, which is the key target for novel antiviral drugs, to ascertain its inhibitory effects based on binding affinity. It proved effective to inhibit the viral spike protein [135], promising to be an interesting phytochemical alternative therapy for COVID-19 and representing a natural compound with antiviral activity and molecular docking techniques. A summary of some of the studies discussed in Section 6.1, Section 6.2, Section 6.3, Section 6.4, Section 6.5 and Section 6.6 and their major findings regarding the biological activity of thyme are shown below in Table 3.

## 7. Innovative Perspective on Thyme

With both thymol and thyme essential oil having been therapeutically used for a long time as an expectorant, anti-inflammatory, anticancer, antibacterial, antiseptic, and antiviral, the current search for new directions in biological or pharmacological activities looks very promising. Likewise, the new formulations of thyme, such as nanogels and microemulsions, can be beneficial in medicinal practice and may create surplus opportunities for better use. In this regard, and to get the best possible from this rich herb, innovative approaches and a systematic method to experimentation with thyme are warranted. This includes establishment of a standard methodology for molecular and mechanistic studies, in addition to better transfer of the data obtained by experimentation into clinical trials, which are still not numerous for this herb. To be able to take full advantage of thyme and its ingredients and completely process them, high quality, advanced phase, randomized, placebo-controlled, multi-centered, randomized, and double-blind clinical trials are needed.

In addition to the major health benefits well documented for thyme, along with the investigated pharmacological properties, there are several traits of this herb that need to be further examined. These include but are not limited to antidiabetic potential, dental decay inhibition, antihelminthic (antiparasitic) properties, and skin protection against ultraviolet radiation [136]. The evidence of health benefits of thyme makes it tempting to further investigate its toxicological, pharmacokinetic, pharmacodynamic, and industrial properties especially in the food industry, as well as its interactions with diet, and realizing pharmacologic studies at the genomic and proteomic levels. It is likely that such assessments of value-added properties of thyme shall be of great interest to the scientific community, ethnopharmacology, and nutrition.

## 8. Limitations of the Review

The judicious review of thyme and its biological activities presented in this work is not without limitations. First, since this work is a narrative review rather than a systematic one, it is possible that some studies on thyme may not have been properly identified and included as needed, in order to be representative of the totality of evidence available on this culinary and medicinal herb. Second, the probability of bias, including influence of the authors’ personal viewpoints, gaps in the literature, probable omission of relevant research, and errors in the translation of data from primary literature about thyme, may all have affected our presented data. Third, with the large number of studies on thyme being reviewed, the quality of the available literature is expected to be heterogeneous, affecting our judgement. An ongoing inquiry on thyme in a more systematic fashion and more critical analysis of the available data is anticipated to circumvent these limitations.

## 9. Conclusions and Future Perspectives

The current status of knowledge regarding thyme depicts a wide plethora of nutritional and therapeutic benefits and provides powerful recommendations for future research directions. With many previous valuable papers reviewing this WEP [136,137,138,139,140], and many yet expected to come, research on thyme remains ongoing with positive anticipation. The widespread use of thymol and thyme essential oil in the food and healthcare industry is quite favorable, but further research and analyses are needed. With increasing demands on food security and medication supply across societies, especially with the COVID-19 pandemic and the brink of a humanitarian disaster in Ukraine, there is a necessity to consider alternative natural compounds as replacements to existing ones. In thyme research, more nutritional and pharmacological investigations should be considered to prove the current findings, and upcoming scientific enquiry should capture future success in the dietetic and clinical benefits of this legendary herb.

## Figures and Tables

**Table 1:** Chemical composition of thyme essential oils [16].

Component	Formula	Relative Concentration (%)
3-Hexanol	C_6_H_12_O	0.10
α-Tujene	C_10_H_16_	1.52
α-Pinene	C_10_H_16_	1.31
Camphene	C_10_H_16_	0.75
Sabinene	C_10_H_16_	0.84
3-Otenol	C_8_H_16_O	0.36
3-Otanone	C_8_H_16_O	0.20
Β-Myrcene	C_10_H_16_	0.67
3-Otanol	C_8_H_18_O	0.21
α-Pellandrene	C_10_H_16_	0.10
δ-3-Carene	C_10_H_16_	0.11
α-Terpinene	C_10_H_16_	2.36
ρ-Cymene	C_10_H_14_	7.61
Sylvestrene	C_10_H_16_	0.34
1,8-Cineol	C_10_H_18_O	0.57
cis-Oimene	C_10_H_16_	0.22
β-Oimene	C_10_H_16_	0.20
ɤ-Terpinene	C_10_H_16_	9.50
cis-Sabinene	C_10_H_8_O	0.10
Thymol	C_10_H_14_O	54.26
Carvacrol	C_10_H_14_O	4.42
Octadienoic acid	C_18_H_12_O	0.10
Geranic acid	C_10_H_16_O_2_	0.30

**Table 2:** The in-depth nutritional profile of *Thymus vulgaris*.

Principle	Nutrient Value per 100 g of Fresh Leaves	Percentage of RDA
Niacin	1.824 mg	11%
Pantothenic acid	0.409 mg	8%
Pyridoxine	0.348 mg	27%
Riboflavin	0.471 mg	36%
Thiamin	0.48 mg	4%
Vitamin-A	4751 IU	158%
Vitamin-C	160.1 mg	266%
Sodium	9 mg	0.5%
Potassium	609 mg	13%
Calcium	405 mg	40.5%
Iron	17.45 mg	218%
Magnesium	160 mg	40%
Manganese	106 mg	15%
Zinc	1.81 mg	16.5%
Carotene-β	2851 mg	-

RDA: Recommended Daily Allowance; -: not estimated.

**Table 3:** Examples of some cited studies on the biological activities of thyme with main results and references.

Biological Activity of Thyme	Major Findings	Reference
Antioxidant	Use of waste thyme extract for preventing the formation of lipid oxidation products in oil in water emulsions, constituted by diverse proportions of wheat and almond oils	[34]
	Antioxidant efficacy of thymol and carvacrol in microencapsulated walnut oil triacylglycerols	[39]
	Effects of thymol and carvacrol on sperm quality and oxidant/antioxidant balance in rats	[41]
	Effectiveness of thymol on the growth performance, antioxidant status of the meat and the immunity of *Nile tilapia* fingerlings, *Oreochromis niloticus*	[43]
Anti-inflammatory	Use of unfractionated essential oil from *Thymus vulgaris* to reduce neutrophil infiltration during an inflammatory response in zebrafish embryos	[45]
	Effectiveness of thyme extract oils in reducing the production and gene expression of proinflammatory mediators and increasing anti-inflammatory IL-10 cytokine secretion in activated macrophages	[48]
	Ability of Greek *Thymus vulgaris* oil and water extracts to detoxify lipopolysaccharide-induced inflammation	[49]
	In vivo anti-inflammatory activities of *Thymus vulgaris* essential oils by significantly reducing carrageenan-induced paw edema in mice	[52]
Anticancer	Anticancer activities of *Thymus vulgaris* L. in experimental breast carcinoma in vivo and in vitro	[68]
	Cytotoxic, genotoxic, apoptotic, and reactive oxygen species (ROS)-generating effects of carvacrol on gastric adenocarcinoma in vitro	[72]
	Chemopreventive effect of thymol against dimethylhydrazine and/or high fat diet-induced colon cancer in rats	[74]
	Effectiveness of carvacrol in inhibiting cell proliferation and migration in non-small cell lung cancer cells	[80]
Antibacterial	Effectiveness of thyme essential oil against Staphylococcus aureus and *Klebsiella pneumoniae*	[90]
	Use of thyme oil nano-emulsions aided by ultrasound to decontaminate the surface of cherry tomatoes against *Eschericia coli* O157:H7	[92]
	Use of thyme essential oil to increase susceptibility to colistin in Nosocomial *Acinetobacter baumannii* and *K. pneumoniae*	[99]
	Bacteriostatic and biofilm inhibitory properties of thyme nanogel against genetically identified skin bacterial clinical isolates (*Pseudomonas stutzeri*, *Enterococcus faecium* and *Bacillus thuringiensis*)	[105]
Antifungal	Fungistatic and fungicidal activity of thyme essential oil against *Candida albicans*	[112]
	Activity of thyme oil and thymol alone or in combination with antifungal drugs as antibiofilm agents against resistant strains of *C. albicans* and *Candida tropicalis*	[114]
	Antifungal control of thyme essential oil on Aspergillus flavus and reduction in aflatoxin B_1_ production, by exerting changes at the molecular level and inducing significant apoptotic-like cell death	[120]
	Activity of thyme essential oil against clinical dermatophytes from the two primary genera *Microsporum* and *Trichophyton*	[125]
Antiviral	Antiviral activity against herpes simplex virus type 2 (HSV-2) by extracts or essential oil of thyme, via decreasing infectivity of the virus particles	[128]
	Dose-dependent anti-influenza activity of thyme extract in Madin Darby canine kidney (MDCK) and HeLa Ohio cells	[129]
	Active interference with Tat protein in HIV, needed in transcription, by the essential oil of thyme	[131]
	Antiviral efficacy of thyme essential oil against feline coronaviruses in vitro, through inhibiting viral replication and reducing viral titer	[133]
	Inhibitory effect of thymol and carvacrol on the spike protein of SARS-CoV2	[135]
